# Supplementary figures and images for: Ventilation‐induced epithelial injury drives biological onset of lung trauma in vitro and is mitigated with prophylactic anti‐inflammatory therapeutics
Source: Bioeng Transl Med. 2021 Dec 1;7(2):e10271. doi: 10.1002/btm2.10271 (PMC9115701; doi:10.1002/btm2.10271)

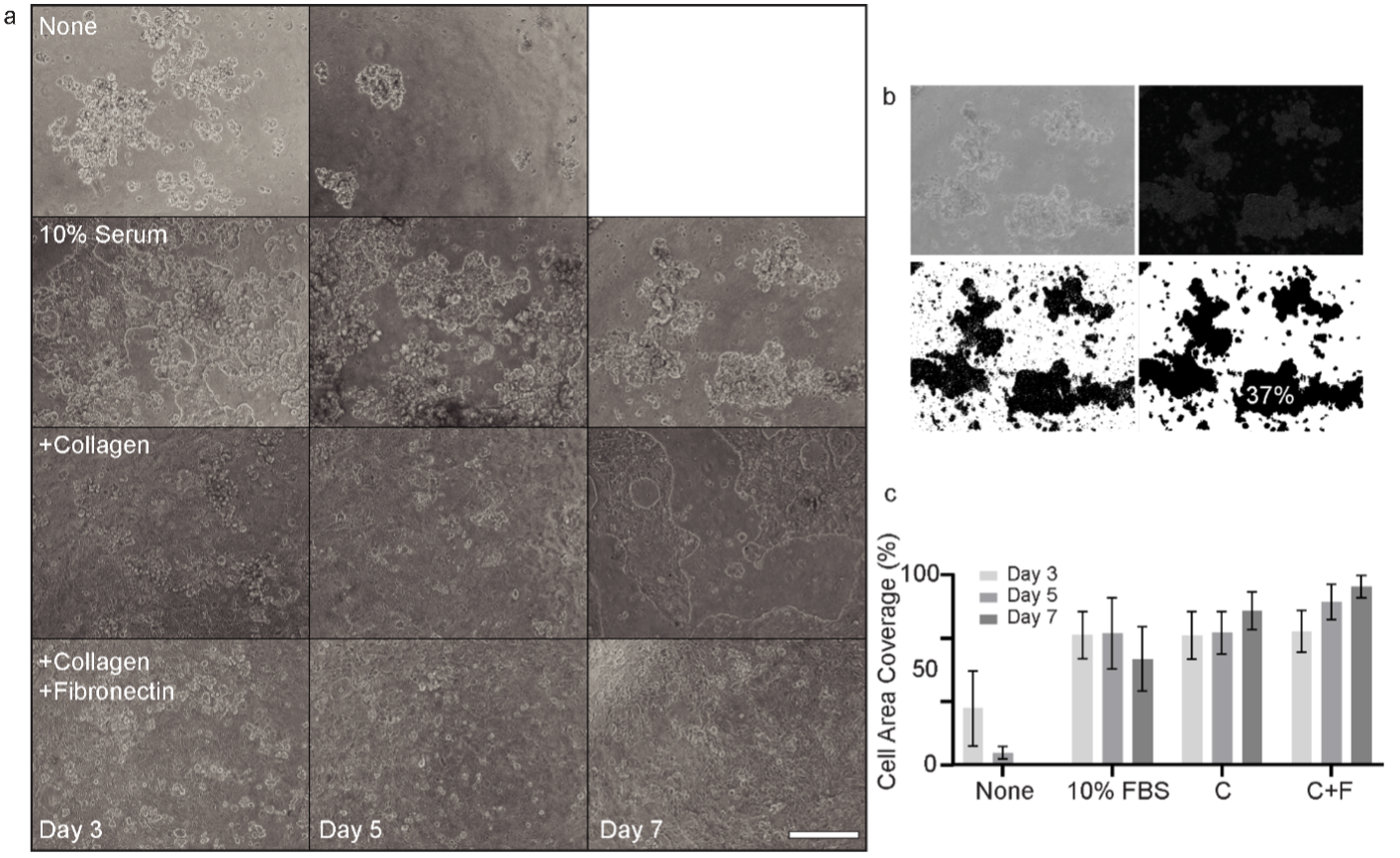

Supplement: Supplementary file 1 — Figure S1: Model lumen coating study for optimal Calu‐3 culture conditions. (a) Bright‐field microscopy images of Calu‐3 bronchial epithelial cells inside a six‐well plate covered with PDMS at 3, 5, and 7 days following initial seeding directly on a PDMS surface, that is, no coating (top row), with 10% fetal bovine serum (FBS) (second row), with 1% v/v collagen (third row) and with a combination of 1% v/v collagen and 1% v/v fibronectin (bottom row). No images were taken after seven days with no coating due to the absence of any remaining live cells. (b) A representative image is shown undergoing four segmentation processing steps used to quantify the area covered by cells in each image (ImageJ, National Institutes of Health). (c) Plot summarizing the results of the study for each coating type at three imaging time points. Error bars signify standard deviation (N = 5) [file BTM2-7-e10271-s001.png]
