# Supplementary material for: Ventilation‐induced epithelial injury drives biological onset of lung trauma in vitro and is mitigated with prophylactic anti‐inflammatory therapeutics
Source: Bioeng Transl Med. 2021 Dec 1;7(2):e10271. doi: 10.1002/btm2.10271 (PMC9115701; doi:10.1002/btm2.10271)
Supplement: Supplementary file 2 — Figure S2: Bronchial epithelial cell line (Calu‐3). Stained confocal microscopy images showing the development of a confluent monolayer when cultured on top of collagen and fibronectin‐coated surface. Occludin (top left) stained green, an integral protein localized at the epithelial cells' tight junctions. F‐actin (bottom left) stained red, a major component of the cytoskeleton. The second column features nuclei stained with blue DAPI. Third column bright‐field microscopy. The last column shows all images as combined overlays characterizing the epithelial monolayer Figure S3: Tomographic particle image velocimetry (TPIV) experimental set‐up comprising an Nd:YLF high‐powered laser, four high‐speed cameras, auxiliary optics, and a phantom PDMS model perfused by a linear motor and syringe. A calibration plate is used for mapping the x‐y‐z world point to the camera chips, and knife edges provide a well‐defined border for uniformly illuminating the measurement volume. Note that in place of the endotracheal tube (ETT) used in the clinically replicated ventilation in vitro experiment, a nylon tube to tube connector is used to prevent leaks Figure S4: Instantaneous 3D velocity vector fields shown at various time points during a ventilation cycle, measured using tomographic particle image velocimetry (TPIV) Figure S5: Computational fluid dynamics (CFD) setup. (a) The model geometry is meshed with tetrahedral cells in ANSYS ICEM and then transformed into polyhedral meshes in ANSYS Fluent. The mesh is refined at the main carina as well as at the secondary bifurcation zones. In the top enlarged inset, a perpendicular cross‐section displays the mesh grid in the upper portion of the trachea, whereas in the bottom inset, an enlarged view of the carina shows increased mesh refinement. In (b), the non‐dimensional mean velocity contours are plotted at several orthogonally‐sliced planes for a representative ventilation case (α=2) and in vector format displayed at the same locations sh [file BTM2-7-e10271-s002.docx]

**Ventilation-induced epithelial injury drives biological onset of lung trauma *in vitro* and is mitigated with prophylactic anti-inflammatory therapeutics**

Eliram Nof^1^, Arbel Artzy-Schnirman^1^, Saurabh Bhardwaj^1^, Hadas Sabatan^1^, Dan Waisman^2,3^, Ori Hochwald^2,4^, Maayan Gruber^5,6^, Liron Borenstein-Levin^2,4^, Josué Sznitman^1^

[1]Faculty of Biomedical Engineering, Technion - Israel Institute of Technology, Haifa, Israel

[2]Faculty of Medicine, Technion - Israel Institute of Technology, Haifa, Israel

[3]Department of Neonatology, Carmel Medical Center, Haifa, Israel

[4]Department of Neonatology, Ruth Rappaport Children's Hospital, Rambam Healthcare, Haifa, Israel

[5]Azrieli Faculty of Medicine, Bar-Ilan University, Safed, Israel

[6]Department of Otolaryngology-Head and Neck Surgery, Galilee Medical Center, Nahariya, Israel

## Supplementary materials


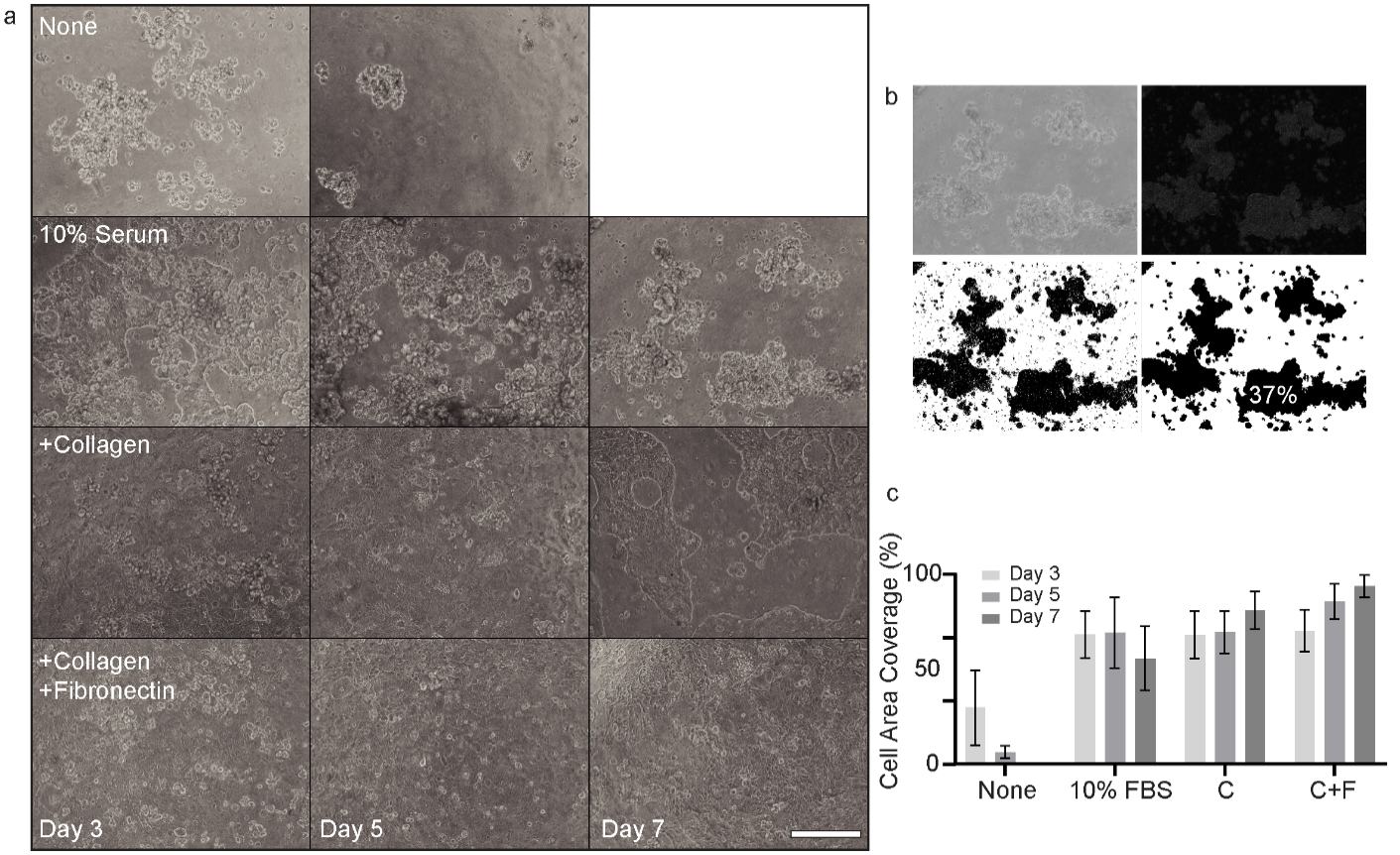


**Supplementary Figure 1**: Model lumen coating study for optimal Calu-3 culture conditions. (**a**) Bright-field microscopy images of Calu-3 bronchial epithelial cells inside a six-well plate covered with PDMS at three, five, and seven days following initial seeding directly on a PDMS surface, i.e., no coating (top row), with 10% fetal bovine serum (FBS) (second row), with 1% v/v collagen (third row) and with a combination of 1% v/v collagen and 1% v/v fibronectin (bottom row). No images were taken after seven days with no coating due to the absence of any remaining live cells. (**b**) A representative image is shown undergoing four segmentation processing steps used to quantify the area covered by cells in each image (ImageJ, National Institutes of Health). (**c**) Plot summarizing the results of the study for each coating type at three imaging time points. Error bars signify standard deviation (N=5).


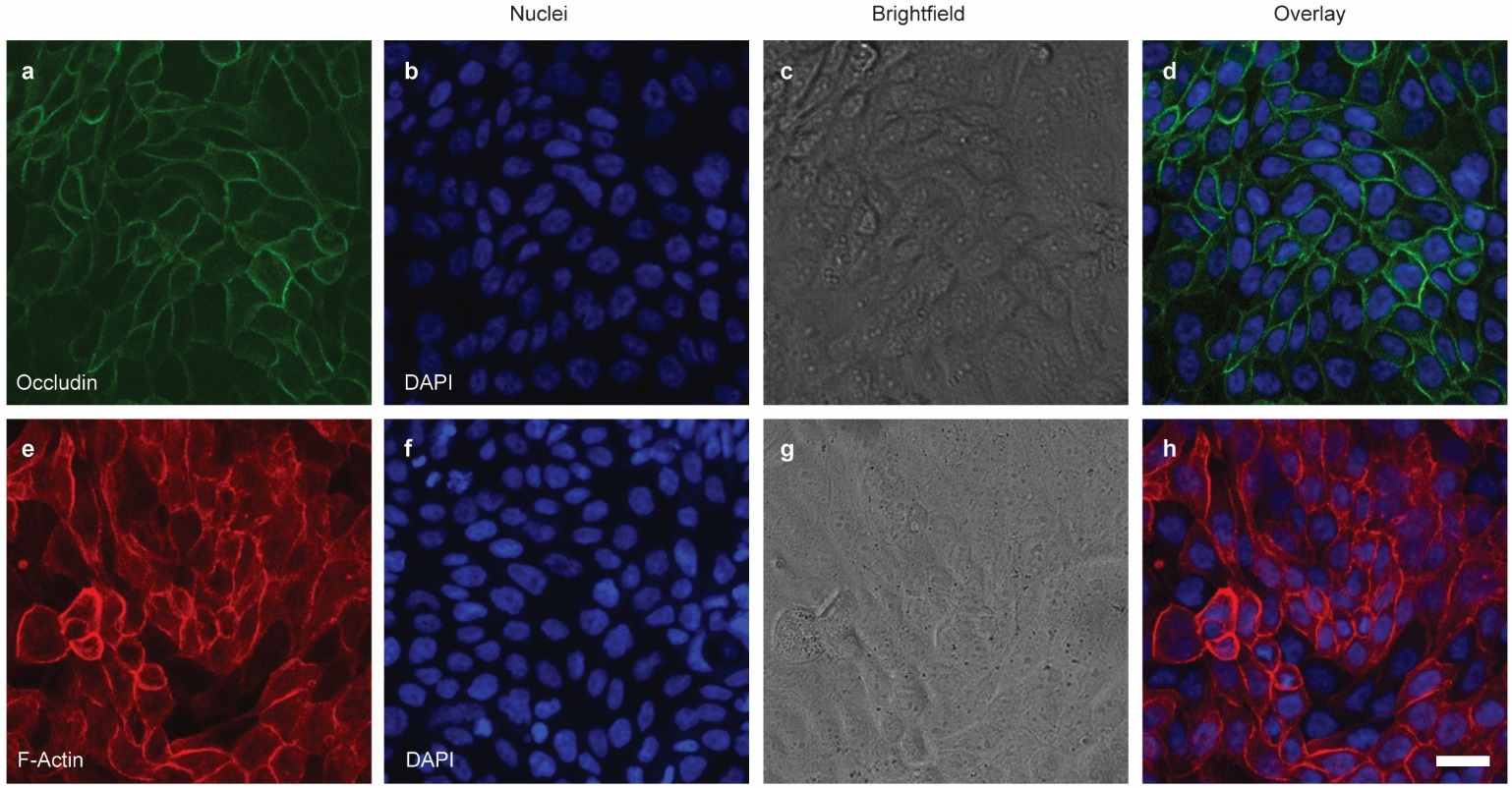


**Supplementary Figure 2**: Bronchial epithelial cell line (Calu-3). Stained confocal microscopy images showing the development of a confluent monolayer when cultured on top of collagen and fibronectin-coated surface. Occludin (top left) stained green, an integral protein localized at the epithelial cells' tight junctions. F-actin (bottom left) stained red, a major component of the cytoskeleton. The second column features nuclei stained with blue DAPI. Third column bright-field microscopy. The last column shows all images as combined overlays characterizing the epithelial monolayer.


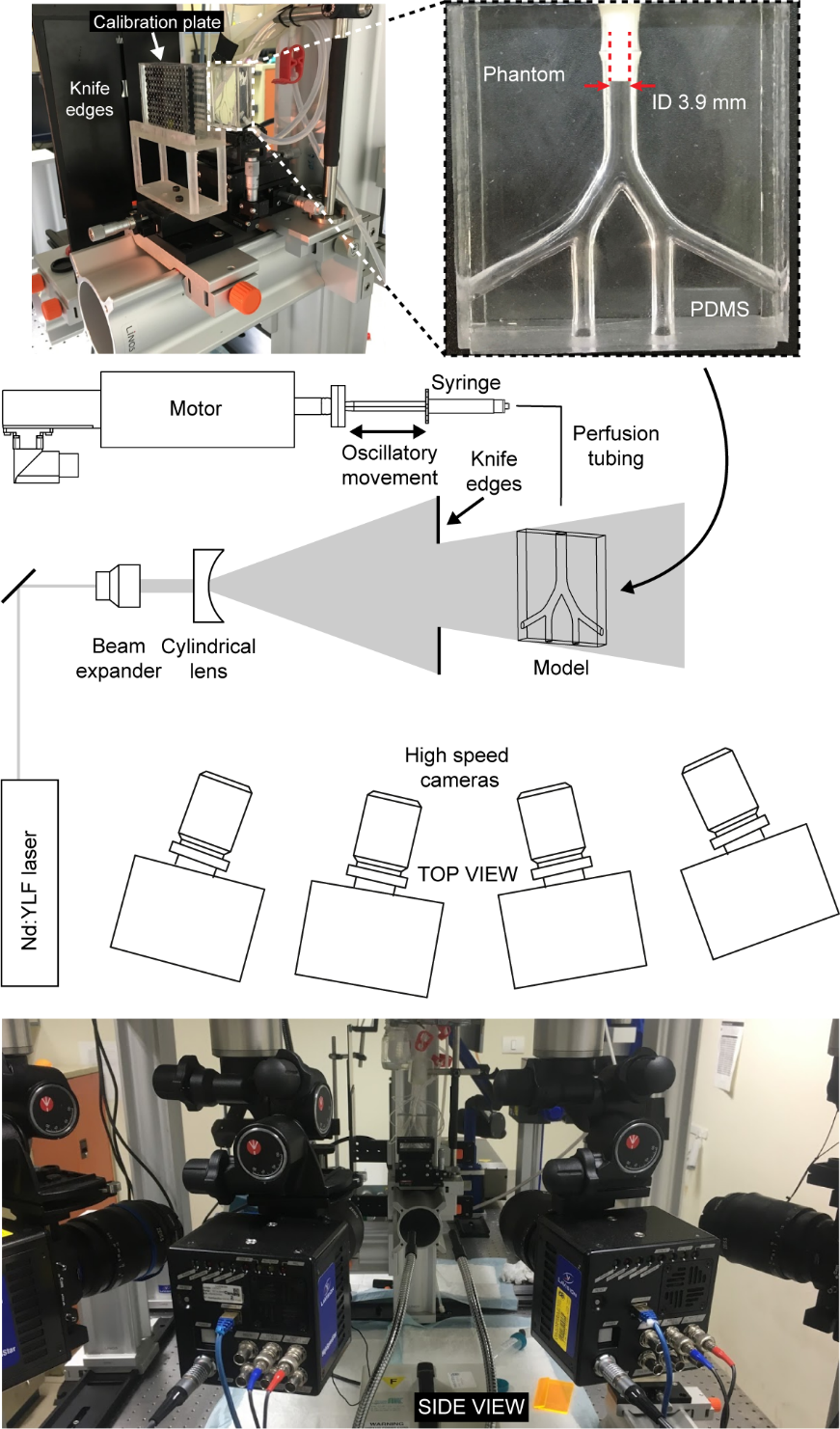


**Supplementary Figure 3**: Tomographic particle image velocimetry (TPIV) experimental set-up comprising an Nd:YLF high-powered laser, four high-speed cameras, auxiliary optics, and a phantom PDMS model perfused by a linear motor and syringe. A calibration plate is used for mapping the x-y-z world point to the camera chips, and knife edges provide a well-defined border for uniformly illuminating the measurement volume. Note that in place of the endotracheal tube (ETT) used in the clinically replicated ventilation *in vitro* experiment, a nylon tube to tube connector is used to prevent leaks.


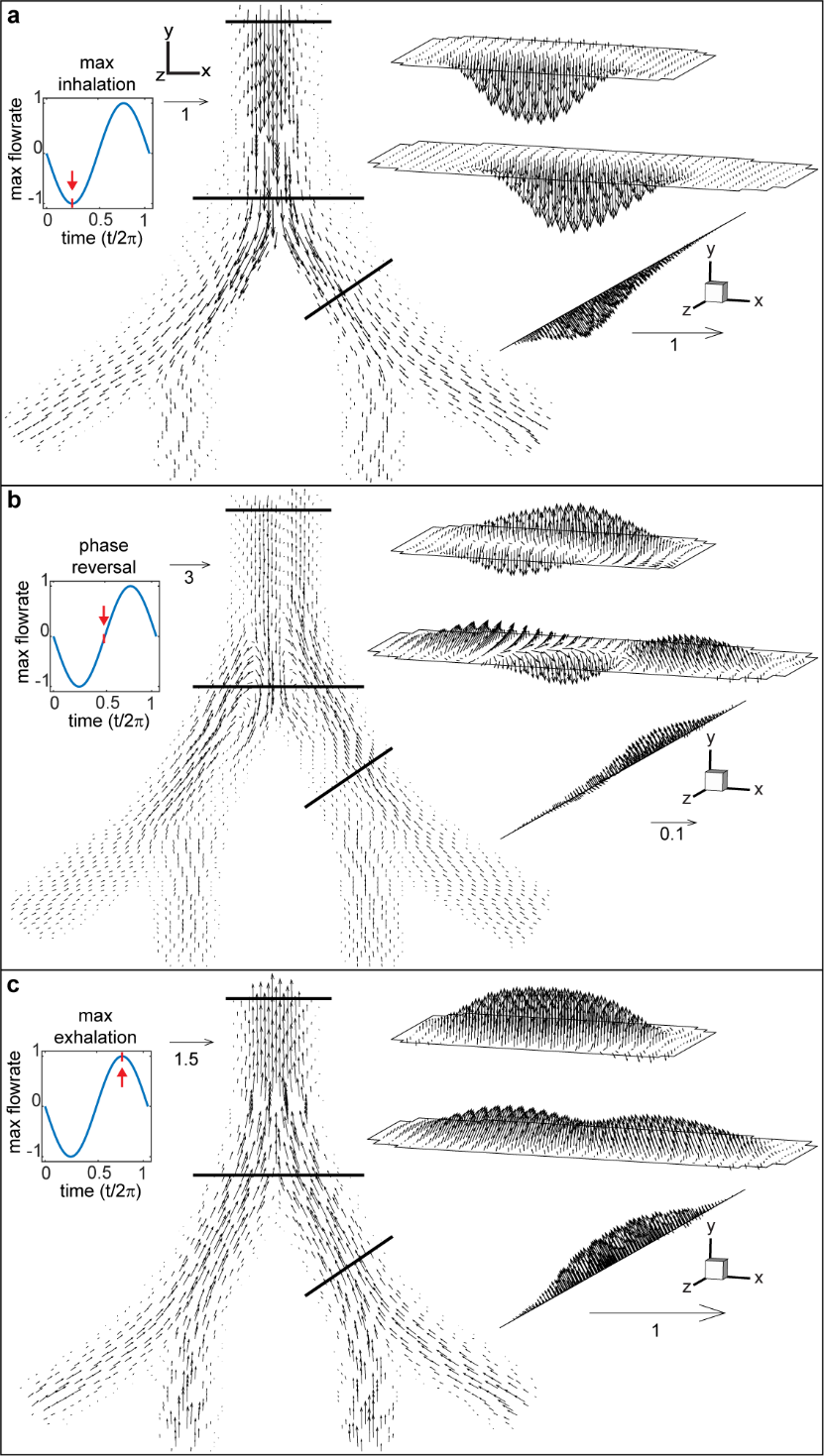


**Supplementary Figure 4**: Instantaneous 3D velocity vector fields shown at various time points during a ventilation cycle, measured using tomographic particle image velocimetry (TPIV).


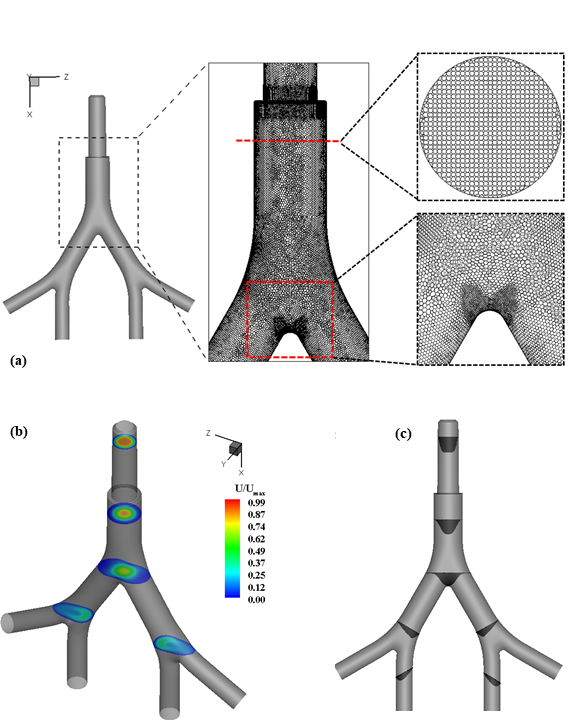


**Supplementary Figure 5**: Computational fluid dynamics (CFD) setup. (**a**) The model geometry is meshed with tetrahedral cells in ANSYS ICEM and then transformed into polyhedral meshes in ANSYS Fluent. The mesh is refined at the main carina as well as at the secondary bifurcation zones. In the top enlarged inset, a perpendicular cross-section displays the mesh grid in the upper portion of the trachea, whereas in the bottom inset, an enlarged view of the carina shows increased mesh refinement. In (**b**), the non-dimensional mean velocity contours are plotted at several orthogonally-sliced planes for a representative ventilation case ($\alpha=2$) and in vector format displayed at the same locations shown in (**c**).


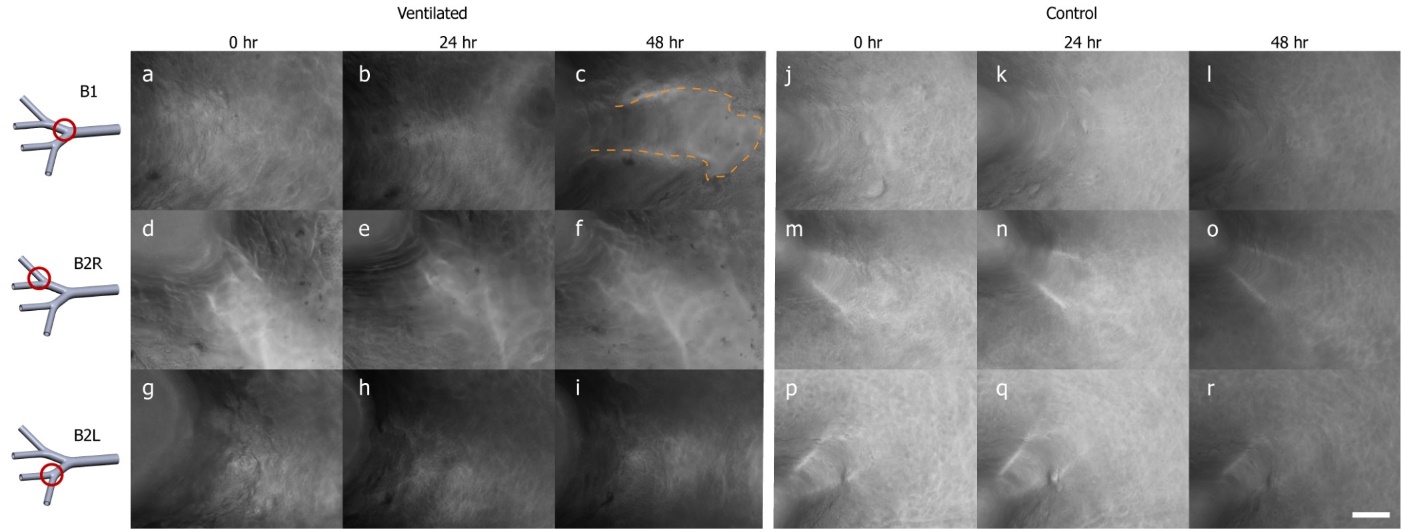


**Supplementary Figure 6**: Cell detachment localized in the high-stress region at first bifurcation appears 48 h after ventilation exposure. Bright-field microscopy images of live cells in the same representative ventilated (a-i) and control (j-f) models compare epithelial cell monolayer integrity at three regions of interest and three time points following ventilation exposure. The regions are the three bifurcations of the model labeled B1 for the first bifurcation(top row; a-c, j-l), B2R for the second bifurcation on the right (second row; d-f, m-o), and B2L for the second bifurcation on the left (bottom row; g-i, p-r) are shown at three time points following ventilation exposure: 0 (i.e., immediately after), 24 h and 48 h. Localized cell detachment was seen only in ventilated models and only at the first bifurcation, highlighted with a dashed line in (c) appearing 48 h following exposure. The scale bar for all images is 100 µm.


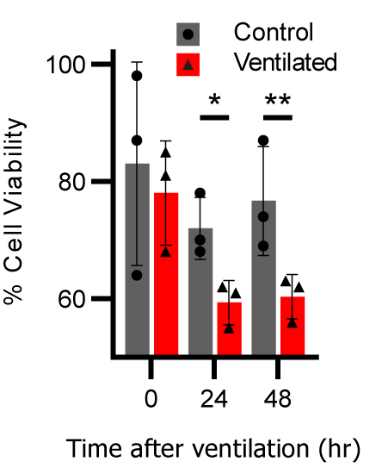


**Supplementary Figure 7**: Cell viability assay. Cell viability was measured by 10% alamarBlue-supplemented culture medium for 2 h in models analyzed three times over 48 hours following the ventilation exposure. Immediately after the exposure (i.e., at time 0 hr), near 100% cell viability is measured, indicating metabolic activity has not been affected in either control or ventilated groups. At 24 h and 48 h, cell viability is reduced by $\sim$40% in the ventilated group while remaining near baseline levels in the control group.
